# Occupational and environmental pesticide exposure and associated health risks among pesticide applicators and non-applicator residents in rural Ethiopia

**DOI:** 10.3389/fpubh.2022.1017189

**Published:** 2022-12-01

**Authors:** Roba Argaw Tessema, Károly Nagy, Balázs Ádám

**Affiliations:** ^1^Department of Public Health and Epidemiology, Faculty of Medicine, University of Debrecen, Debrecen, Hungary; ^2^Doctoral School of Health Sciences, University of Debrecen, Debrecen, Hungary; ^3^Department of Environmental Health Sciences, College of Health and Medical Sciences, Haramaya University, Harar, Ethiopia; ^4^Institute of Public Health, College of Medicine and Health Sciences, United Arab Emirates University, Al Ain, United Arab Emirates

**Keywords:** pesticide exposure, health risk, occupational health, applicators, residents, preventive measures

## Abstract

Intensive pesticide use increased concern about the potential acute and chronic health effects of pesticides in general and among applicators in particular. This study aims to explore occupational and environmental pesticide exposure and health risks among pesticide applicators and residents. A community-based cross-sectional study was conducted involving 1,073 individuals. We examined the health effects potentially attributable to pesticide exposure using regression to estimate prevalence ratios (PR). A higher proportion of good knowledge of pesticides [75 vs. 14%; APR = 1.542 (1.358–1.752), *p* < 0.001] and a higher mean score of perceived health risk of pesticide use [4.21 vs. 3.90; APR = 1.079 (1.004–1.159), *p* < 0.05] were observed among applicators than residents. A significantly higher proportion of applicators experienced health effects presumably related to pesticide exposure among themselves (36%) than residents (16%), and a higher proportion of them used prescribed drugs in the past 12 months [51 vs. 32%; APR = 1.140 (1.003–1.295), *p* < 0.05]. Skin irritation, shortness of breath, cough, and dizziness were more likely reported by applicators than residents. Perceived toxicity of currently applied pesticide products, mix pesticides without gloves, regularly maintain and wash sprayer tank after application, occurrence of an incidental splash during mixing and application, and using home-based care after experiencing a symptom presumably due to pesticide exposure were significantly associated with health effects among applicators. Use of face mask and visiting health facility when experiencing a symptom presumably due to pesticide exposure were significantly positively correlated with attending training on the health risks and use of pesticides. A substantial proportion of applicators reported improper use of preventive measures and methods of pesticide waste disposal. These observations point out that applicators can face high health risks of occupational pesticide exposure in Ethiopia. Even trained applicators pursued poor preventive practices; hence, comprehensive practice-oriented in-depth training focusing on safety precautions and proper use of personal protective equipment, and provision of adequate pesticide waste disposal means are crucial interventions.

## Introduction

Pesticides contribute substantially (i) to increase yield quantity and quality through crop protection against weeds, diseases, and pests (1); (ii) to protect humans against the vectors of infectious diseases, like malaria, leishmaniasis, typhus, plague, and dengue (2); and (iii) to control weeds, pathogens, insect and rodents in domestic settings (3). Nevertheless, inappropriate or excessive use of pesticides poses a significant risk to non-target organisms, including humans, and to the environment (4). Therefore, balancing the need for chemically synthetic pesticides with the risk to human health and the environment becomes a critical challenge globally in the future. Malpractice related to pesticides is a major public health problem particularly in low and middle-income countries where enforcement of regulations on pesticide use and public health tend to be less strict and health surveillance systems less effective (5). The health effects of pesticide exposure depend on the chemical nature of the pesticide, route of exposure (inhalation, ingestion, or skin absorption), frequency, duration, and intensity of exposure (6). Short-term exposure to high levels of pesticide can lead to acute intoxications, whereas long-term low-dose exposure may induce mostly chronic effects (7).

A recent review of scientific literature and WHO mortality data indicated that about 385 million cases of unintentional acute pesticide poisoning (UAPP) occur globally each year, with around 11,000 fatalities (8). A steep increase from 25 million cases estimated in 1990 (9). Based on the worldwide farming population of about 860 million, 44% of farmers are poisoned by pesticides every year. References to non-fatal UAPP, the highest estimate of UAPP cases was observed in southern and south-eastern Asia, followed by east Africa, where Ethiopia is situated (8). Pesticide self-poisoning accounted for 14–20% of global suicides leading to 110,000–168,000 deaths annually (10). The high frequency of intentional pesticide self-poisoning can partly be attributed to the poor management system of pesticides including highly toxic ones.

Acute pesticide poisoning can cause a range of symptoms in adults and children. Pesticides can induce neurotoxic effects such as headaches, dizziness, confusion, restlessness, muscle twitching, slurred speech, unconsciousness; digestive system effects such as burning sensation in the mouth and throat, excessive salivation, nausea, vomiting, abdominal pain, and diarrhea; respiratory effects such as cough, chest pain and tightness, difficulty with breathing and wheezing; effects involving the skin such as irritation, burning sensation and excessive sweating; and the eyes such as itching, burning sensation, watering and blurred vision (11–13). Pesticides are related to several chronic health effects, including developmental and reproductive effects such as spontaneous abortions, stillbirths, lower birth weights, birth defects, and early neonatal deaths; carcinogenic effects both in children and adults including carcinomas, such as prostate cancer, leukemia and non-Hodgkin lymphomas (13–17). Studies in Ethiopia indicated that the overall prevalence of chronic diseases is 9% (8% men and 10% women), 3.1% diabetes, 3% cardiac disease, 9.3% hypertension, 1.5% asthma, 0.5% epilepsy, 1.7% depression, and 10.7% high total cholesterol. Eighty percentage of the study population had at least one risk factor for chronic disease, and pesticide exposure may contribute to this hidden burden (18, 19).

Due to widespread dispersion, persistence, and bioaccumulation in the environmental compartments, several sub-groups of people (operators, workers, residents, and bystanders) can have different patterns and degrees of exposure and are at varying risk of adverse effects (20). Occupational exposure occurs among pesticide manufacturers, formulators, vendors, transporters, mixers, loaders, operators of application (farmers) and clean-up workers due to their direct involvement in the handling of pesticides. Resident exposure may occur due to pesticide drift or residues in food, drinking water, soil, dust and air, clothing, and direct contact (21).

Agriculture is the mainstay of the Ethiopian economy, constituting over 50% of the gross domestic product (GDP), over 85% of the employment, and earning over 90% of the foreign exchange (22). Due to the intensification and expansion of modern agriculture including commercial horticultural farms, small-scale irrigated farms, large-scale open farms, and cut-flower greenhouses chemical pesticides consumption has shown an almost three-fold increase over the last decade (1,440–4,586 tons from 2001 to 2013, respectively) (23). Studies in Ethiopia indicated that a high quantity of pesticide residues is found in drinking water (24, 25), soil (26), and in food (27, 28) that could cause chronic health risks to the public. In addition, studies showed evidence of pesticide contamination of non-target organisms like soil organisms, fish, bee colonies, and wildlife (26, 29–32).

The low level of education, poor knowledge about pesticide hazards, misperception about pesticide exposure, inappropriate handling, storage, and disposal practices and the poor use of personal protective equipment are the most important barriers to the adoption of self-protective behaviors among farmers in Ethiopia (23, 33). This may be exacerbated by poor extension services, that encompass a range of activities from training individual farmworkers to follow pesticide safety precautions, use personal protective equipment properly and develop self-protective behavior to advise the agricultural companies to ensure appropriate management, effective and safe use of agrochemicals including pesticides (34, 35).

There is a great concern about the negative impact of pesticides on human health and on the environment in Ethiopia due to their wide distribution and potentially harmful effects. Consequently, to protect applicators' health and the environment, and to improve the sustainability of chemical pest control, increased knowledge on applicators' and residents' health risks due to occupational and environmental pesticide exposure is necessary. Evidence from studies conducted by Tessema et al. (35) and Mormeta (34) indicated that extension officers, who are theoretically and practically expected to be experts in providing appropriate advice, educating proper and safe handling practices of pesticides to applicators and users, have insufficient knowledge about the pesticides themselves (34, 35), which may predispose the applicators to higher risk of pesticide exposure. Hence, knowledge about the prevalence of pesticide exposure and related health risk for applicators and residents is crucial for planning effective interventions. To our knowledge, there are scanty studies investigating and comparing the risk of occupational and non-occupational pesticide exposure and related health risks among applicators and residents in Ethiopia. Therefore, the aim of this study is to explore and compare the health risks of occupational and non-occupational exposure to pesticides among pesticide applicators and residents in Ethiopia, which enables us forward concrete and actionable recommendations for mitigating harm associated with pesticide application.

## Materials and methods

### Study design

**Figure 1:**
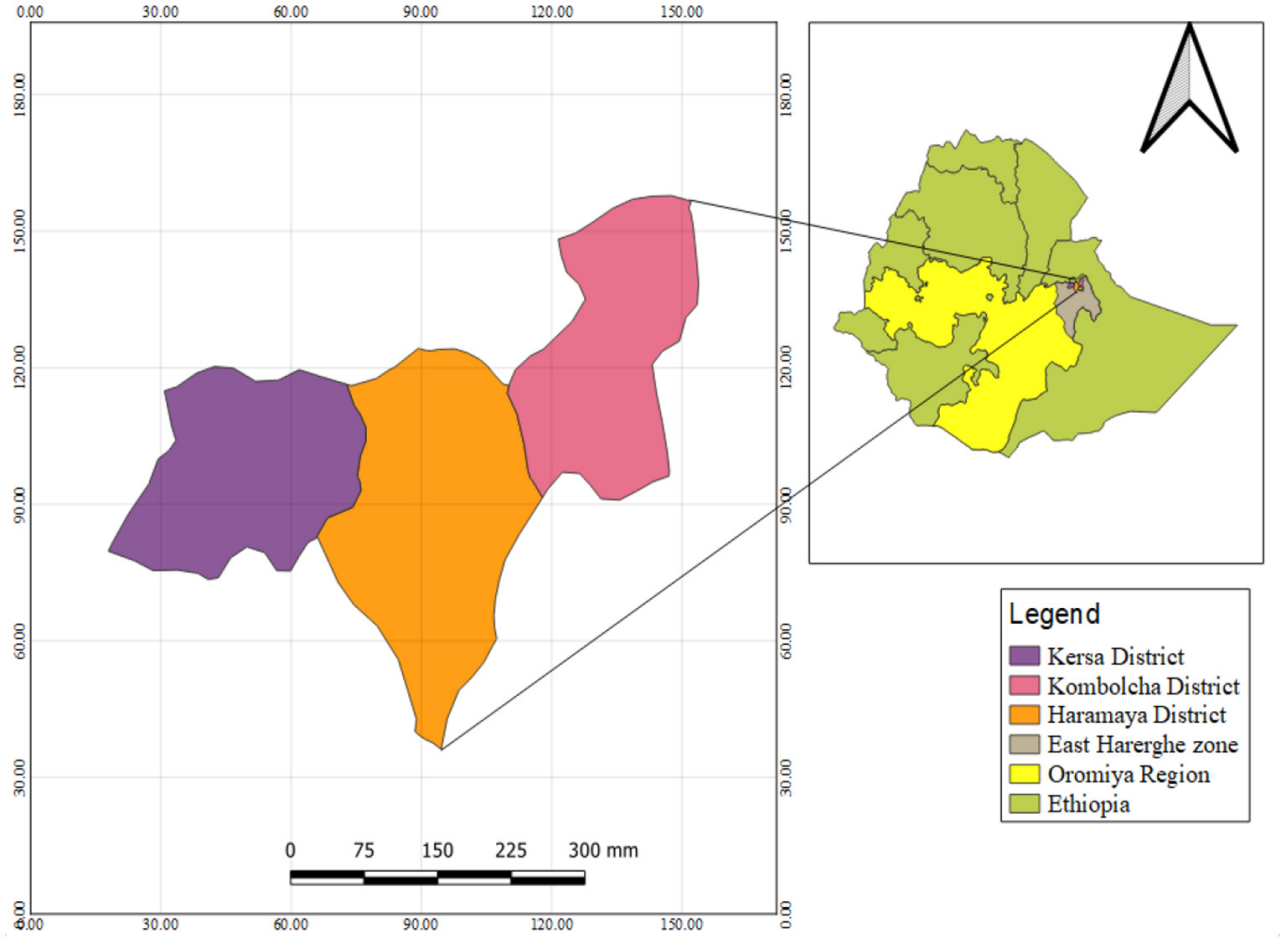
Map of the study area, Kersa, Kombolcha, and Haramaya districts, East Hararge Zone, Oromiya Region, Ethiopia.

A community-based cross-sectional survey was conducted from 26 April to 31 August 2021 to investigate the health risks of occupational and environmental pesticides exposure and associated health risks among pesticide applicators compared to residents in Ethiopia. The study took place in East Hararge Zone within the region of Oromiya, Ethiopia. The study was conducted in the East Hararge Zone within the region of Oromiya, encompassing a total of 3,286,338 population situated in 17,935.40 km^3^. Out of 18 districts of the zone, the study was conducted in three districts, where heavily agricultural activities conducted and extensive pesticide use, namely in Kersa [a total of 43,191 households situating in 35 kebeles (“kebele” is smallest administrative units in Ethiopia)], in Haramaya (a total of 70,406 households dwelling in 38 kebeles) and in Kombolcha (a total of 35,611 households residing in 20 kebeles) (36). A total of 15,908 households in ten selected kebeles of the three included districts served as the source population (Figure 1). Individuals in the selected households who applied pesticides in their farmland were considered as pesticide applicators. They were farm workers who were involved in pesticide handling including mixing, loading, repairing, cleaning, and operating application machinery (spray tanks). Individuals in the selected households, who were not involved in pesticide application and farm activities, were considered residents (37, 38). In the same household, an applicator or a resident would be selected.

### Sampling

The sample size was determined by using single population proportion formula (39) with a z value corresponding to 95% confidence level (z = 1.96), d = margin of error stated as a proportion of 0.05 at a 95% confidence level (d = 0.05), p_a_ = proportion of pesticide applicators who experienced acute pesticide intoxication due to pesticide application in their farms land; p_r_ = proportion of residents who experienced health problem by living in the proximity (< 5 km) of pesticide treated farm land. From a previous study in Ethiopia, p_a_ was taken as 0.56 for applicators and for residents p_r_ was taken as 0.23 (40). For applicators, this gave a sample size of 740 with a design effect of 2. For residents, it gave a sample size of 268. Therefore, the minimum sample size to attain statistical significance was 1,008 (740 applicators and 268 residents) subjects. A design effect of 2 was used to obtain the sample size needed under the multistage sampling design.

A multistage sampling technique was used to select districts, kebeles, households, applicators, and residents. The sampling consisted of four steps. In the first step, three districts were selected based on the extent of pesticide use and agricultural activities from the 18 districts of the East Hararge Zone, Oromiya Region Ethiopia. In the second step, probability proportional to size (PPS) sampling was used to calculate the number of kebeles of each district after the total number of kebeles in three districts was identified. Thirdly, the number of households was determined by PPS of each kebele after the total number of households in selected kebeles was identified. In the last step, every 14th household was contacted from the randomly selected kebeles of the study area. To determine the starter household, lottery method was used. From each contacted household, an eligible pesticide applicator or a resident (age >18 years who can properly communicate) was interviewed. If there was no pesticide applicator in the household, a randomly selected eligible dweller was interviewed as a resident. If there were two applicators in one household, the one who more recently applied pesticide was interviewed. Only an applicator or a non-applicator resident was interviewed in a selected household. The process was continued until more individuals were interviewed than the minimum required sample size calculated.

### Data sources and collection, variables

Data were collected by questionnaire-based face-to-face interviews. Three interviewers with previous experience in data collection were employed after training on how to administer the questionnaire. Field supervision was made by the principal investigator (RAT) during data collection daily. Data collection included the administration of questionnaires to pesticide applicators and residents selected for the study during the study period. The information was collected from each participant after signing the written consent form. Data collections from applicators and residents were done simultaneously. The survey questionnaire was designed based on questionnaires previously applied in published studies elsewhere (41–43) with slight modifications to meet the current study objectives (Supplementary file). The questionnaire was prepared in English and translated into Afan Oromo, and then back to English to ensure its consistency. The structured and pre-tested questionnaire comprised of 66 closed and open-ended questions. The questionnaire collected information on seven domains: (1) socio-demographic (gender, age, marital status, income level, family size, and educational status) and lifestyle factors (past and present smoking history, alcohol, and Khat consumption status) measured by multiple-choice questions (MCQs); (2) knowledge about pesticide products, mode of exposure and effects (health risks and environmental impacts associated with pesticide use) measured by dichotomous response followed by open-ended response; (3) attitude related to pesticide use and health risks of pesticide exposure (effectiveness of pesticide management systems, perceived health risk of pesticide exposure, perception of pesticide residue pollution of environmental compartments, perception about PPE use and training in reducing health risk of pesticide exposure, etc.) assessed by five-item psychometric Likert scale anchored from strongly disagree to strongly agree; (4) diagnosed health problems potentially related to pesticide exposure measured by dichotomous response followed by open-ended response; and (5) experienced symptoms apparently related to pesticide poisoning assessed by five-item psychometric Likert scale anchored from never happen to happen always; (6) occupational pesticide use, exposure history and experienced practice explored by MCQs, and (7) applied preventive measures (use of PPE and its availability, training about health risks of pesticides, actions taken after pesticide poisoning, personal hygiene practice, safety practices regarding equipment calibration, tank mixing, application techniques, disposal of leftover pesticide and evidence of pesticide spillage, etc.) assessed by mixed questions (MCQs, dichotomous, Likert scale and open-ended response). The first 40 questions were answered by both applicators and residents. From question 40, only participants who currently applied pesticides in their farmland continued to complete the questionnaire.

To measure applicators' and residents' knowledge related to pesticide products, routes of pesticide exposure, environmental problems associated with pesticide use, and health effects presumably due to pesticide exposure, the following questions were addressed to applicators and residents: (1) Do you know pesticides product used by their name? (scored 0 for no response; 1 for yes), and list at least one pesticide product (score 0 for no correct response; 1 for >1 correct responses); (2) Do you know routes of pesticide exposure? (scored 0 for no response; 1 for yes), and list at least one route of exposure product (score 0 for no correct response; 1 for >1 correct responses); (3) Do you know environmental problems related to pesticide use? (scored 0 for no response; 1 for yes), and list at least one problem (score 0 for no correct response; 1 for >1 correct responses); (4) Do you know health effects pesticide exposure can induce? (scored 0 for no response; 1 for yes), and list at least one health effect (score 0 for no correct response; 1 for >1 correct responses). Based on the scores the total knowledge score was calculated. The mean value was used to dichotomize between the poor (scored less than or equal to mean value) and good (scored higher than mean value) knowledge categories.

### Statistical analysis

Comparison of the frequencies of health effects or symptoms potentially attributable to pesticide exposures among pesticide applicators and residents were carried out to determine the level of health risks associated with occupational and environmental pesticide exposure. Data analysis was made using SPSS Version 25 statistical package. Descriptive statistics, such as mean, standard deviation, frequency, and proportion were calculated in univariate analysis. The mean score of knowledge of and attitude toward pesticides was determined. Chi-square tests were used to compare the association between socio-demographic and lifestyle factors, knowledge of and attitude to pesticides, pesticide use and exposure, health and experienced symptoms, and preventive measures among applicators and residents. Using generalized leaner models, a log-binomial regression was computed, and unadjusted prevalence ratios (CPR) and confounder-adjusted prevalence ratios (APR) were calculated to investigate the strength of association between outcome and explanatory variables. The log-binomial regression model produces unbiased prevalence ratio estimates in cross-sectional studies of common outcomes avoiding the overestimation from odds ratios (44, 45). To identify the predictive variables of knowledge and attitude, adjustment for sex, age, marital status, family size, income level, and education level was used. In case of medical conditions, experienced symptoms and health effects, additional adjustment was made for past and present smoking status, frequency of alcohol and Khat consumption. The significance of statistical associations was assured using prevalence ratios with a 95% confidence interval (CI) and *p*-values. Statistical significance was accepted at 5% level.

## Results

### Socio-demographic and lifestyle characteristics

In this study, a total of 1,073 participants (803 applicators and 270 residents) were contacted, and all the contacted eligible persons responded, which gives a 100% response and participation rate. The majority (93% for applicators; 83% for residents) of the respondents were male. Thirty- and thirty-four percent of the respondent's ages ranged from 40 to 49 years, with the mean age of 42 (±10.12 SD) and 41 (±10.18 SD) years for applicators and residents, respectively. Ninety-one and eighty-five percent of the applicators and residents were married, respectively, and 46% of the applicators and 37% of the residents attended tertiary education. Forty–three percent of applicators and 44% of residents, 11% of applicators and 24% of residents, and 87% of applicators and 92% of residents were currently cigarette smokers, consumed alcohol 2–4 times a month and chewed Khat daily, respectively (Table 1).

**Table 1:** Socio-demographic characteristics and lifestyle factors of pesticide applicators and residents in East Hararge Zone, Oromiya Region, Ethiopia.

**Attributes (*n* = 1,073)**	**Options**	**%**	**Applicators**	**Residents**	*p-value
			**(*n* = 803)**	**(*n* = 270)**	
Sex	Male	90.0%	92.5%	82.6%	< 0.001
	Female	10.0%	7.5%	17.4%	
Age (years)	20–29	13.0%	12.1%	15.9%	0.111
	30–39	29.7%	30.4%	27.8%	
	40–49	31.4%	30.4%	34.4%	
	50–59	25.8%	27.1%	21.9%	
Marital status	Married	89.4%	90.8%	85.2%	0.010
	Single	10.6%	9.2%	14.8%	
Family size	Less than or equal to 3	11.6%	13.6%	5.9%	0.001
	Greater than 3	88.4%	86.4%	94.1%	
Under-five child	None	87.8%	87.7%	88.1%	0.808
	1–2	10.3%	10.2%	10.4%	
	>3	2.0%	2.1%	1.5%	
Monthly income	Below average (< 9000 ETB)	10.9%	9.7%	14.4%	0.031
	Above average (>9000 ETB)	89.1%	90.3%	85.6%	
Educational level	Primary (grade 1–8)	19.4%	17.1%	26.3%	0.002
	Secondary (grade 9–12)	36.9%	37.1%	36.3%	
	Tertiary (> 12 grade)	43.7%	45.8%	37.4%	
Current smoking status	Daily	43.2%	42.8%	44.1%	0.014
	Less than daily	35.4%	37.5%	29.3%	
	Not at all	21.4%	19.7%	26.7%	
Past smoking status	Daily	49.8%	53.7%	38.1%	< 0.001
	Less than daily	38.7%	39.7%	35.6%	
	Not at all	11.6%	6.6%	26.3%	
Frequency of alcohol consumption	2–4 times a month	14.1%	10.7%	24.1%	< 0.001
	Monthly or less	17.1%	16.1%	20%	
	Don't drink	68.9%	73.2%	55.9%	
Alcohol consumption on a typical day when drinking	5–6 drinks	7.7%	6.6%	11.1%	< 0.001
	3–4 drinks	11.7%	9.0%	20.0%	
	1–2 drinks	11.6%	11.2%	13.0%	
Consume six or more alcohol drink on one occasion	Monthly	10.7%	6.5%	23.3%	< 0.001
	Less than monthly	10.1%	6.8%	19.6%	
	Not at all	79.2%	86.7%	57.0%	
Khat (Catha Edulis) consumption	Daily	88.1%	86.9%	91.5%	0.046
	Less than daily	11.9%	13.1%	8.5%	

ETB, Ethiopian birr; ^*^p < 0.05 taken as stastically significant.

### Knowledge of and attitude to pesticides

This study indicates that applicators have better knowledge about pesticide products (75% had good overall knowledge, of whom 93.5% cited at least one pesticide product) and routes of exposure (79%) than residents (23 and 30%, respectively) (Table 2). A significantly higher proportion of applicators were knowledgeable about health effects induced by pesticide exposure (79%) than residents (34%). In multivariate analysis, knowledge of pesticide products [APR = 1.429 (1.263–1.617), *p* < 0.001], routes of pesticide exposure [APR = 1.372 (1.221–1.553), *p* < 0.001], environmental problems related to pesticide use [APR = 1.332 (1.186–1.506), *p* < 0.001], health effects induced by pesticide exposure [APR = 1.332 (1.183–1.501), *p* < 0.001] and overall knowledge [APR = 1.542 (1.358–1.752), *p* < 0.001] was significantly more advanced among applicators (Table 2).

**Table 2:** Knowledge of pesticides among pesticide applicators and residents in East Hararge Zone, Oromiya Region, Ethiopia.

**Respondents' knowledge of pesticides**	**Applicators (*N* = 803)**	**Residents^R^ (*N* = 270)**	**Unadjusted PR (95% Cl)**	**Adjusted PR^†^ (95% Cl)**
	**Good% (95% Cl)**	**Good% (95% Cl)**		
Knowledge of pesticide products	75.0 (71.8–77.9)	22.6 (17.7–28.1)	1.427 (1.266–1.609)***	1.429 (1.263–1.617)***
Knowledge of routes of pesticide exposure	79.0 (76.0–81.7)	30.4 (24.9–36.2)	1.373 (1.222–1.542)***	1.372 (1.221–1.553)***
Knowledge of environmental problems related to pesticide use	78.1 (75.1–80.9)	34.1 (28.4–40.1)	1.328 (1.184–1.491)***	1.332 (1.186–1.506)***
Knowledge of health effects pesticide exposure can induce	78.8 (75.8–81.6)	34.1 (28.4–40.1)	1.334 (1.189–1.497)***	1.332 (1.183–1.501)***
Overall knowledge level about pesticide hazards	75.1 (72.0–78.1)	13.7 (9.8–18.4)	1.540 (1.361–1.742)***	1.542 (1.358–1.752)***

^†^Adjusted for sex, age, marital status, family size, income level, and education level; ^R^Reference category; ^***^P < 0.001; PR, Prevalence Ratio; “Good” knowledge proportion of applicators and residents.

Inhalation and dermal contact were the most frequently named routes of pesticide exposure by applicators and residents, respectively; and applicators' knowledge about all routes was significantly higher. Water pollution was the most often named major pesticide-related environmental health problem by both applicators and residents, and all relevant environmental problems were significantly better known among applicators. Asthma and cancer were the most frequently named pesticide-related health effects by applicators and residents, respectively. Applicators were more knowledgeable about four health effects including the most important ones (Table 3).

**Table 3:** Knowledge of routes of exposure, problems that pesticides pose on the environment, and health effects of pesticide exposure among pesticide applicators and residents in East Hararge Zone, Oromiya Region, Ethiopia.

**Characteristics**		**Applicators**	**Residents^R^**	**Unadjusted PR**	**Adjusted PR^†^**
		**(*n* = 803)**	**(*n* = 270)**	**(95% Cl)**	**(95% Cl)**
		**Yes% (95% Cl)**	**Yes% (95% Cl)**		
The routes through which pesticides can enter the human body	Inhalation	70.0 (66.7–73.1)	27.8 (22.5–33.5)	1.330 (1.109–1.595)**	1.332 (1.103–1.505)***
	Oral ingestion	64.9 (61.5–68.2)	27.4 (22.2–33.1)	1.294 (1.079–1.552)**	1.290 (1.141–1.458)***
	Dermal contact	61.3 (57.8–64.7)	30.0 (24.6–35.8)	1.241 (1.035–1.487)*	1.238 (1.095–1.398)***
The major problems of the environment associated with pesticide use	Water pollution	78.7 (75.7–81.5)	31.1 (25.6–37.0)	1.363 (1.138–1.632)***	1.370 (1.215–1.545)***
	Soil contamination	77.5 (74.4–80.3)	29.6 (24.2–35.5)	1.369 (1.143–1.640)***	1.380 (1.223–1.558)***
	Food contamination	72.7 (69.5–75.8)	24.8 (19.8–30.4)	1.384 (1.153–1.660)***	1.391 (1.230–1.573)***
	Air pollution	73.1 (69.9–76.1)	23.7 (18.8–29.2)	1.399 (1.166–1.679)***	1.406 (1.243–1.591)***
	Harm to non-target animals	43.3 (39.9–46.8)	18.1 (13.7–23.3)	1.213 (1.008–1.460)*	1.215 (1.069–1.382)**
Health effects pesticide exposure can induce	Asthma	55.3 (51.8–58.8)	17.8 (13.4–22.9)	1.319 (1.096–1.586)**	1.311 (1.154–1.488)***
	Cancer	52.6 (49.0–56.1)	18.1 (13.7–23.3)	1.291 (1.142–1.460)**	1.294 (1.139–1.469)***
	Diarrhea	38.4 (35.0–41.8)	15.6 (11.4–20.4)	1.197 (1.056–1.358)**	1.182 (1.038–1.346)**
	Allergy	34.6 (31.3–38.0)	14.8 (10.8–19.6)	1.172 (1.033–1.330)**	1.176 (1.032–1.340)**
	Headache	26.5 (23.5–29.7)	13.0 (9.2–17.6)	1.138 (1.001–1.293)*	1.118 (0.979–1.276)
	Diabetes	14.1 (11.7–16.7)	13.0 (9.2–17.6)	1.010 (0.887–1.150)	1.018 (0.890–1.164)
	Nerve disorders	6.8 (5.2–8.8)	0.7 (0.1–2.7)	1.061 (0.925–1.216)	1.057 (0.918–1.218)
	Stomach pain	6.7 (5.1–8.7)	3.0 (1.3–5.8)	1.037 (0.905–1.187)	1.034 (0.898–1.190)
	Liver dysfunction	5.7 (4.2–7.6)	0.7 (0.1–2.7)	1.050 (0.916–1.203)	1.049 (0.911–1.209)
	Infertility	5.4 (3.9–7.1)	4.4 (2.3–7.6)	1.009 (0.882–1.154)	1.010 (0.879–1.162)
	Stroke	5.2 (3.8–7.0)	2.2 (0.8–4.8)	1.029 (0.899–1.179)	1.029 (0.894–1.185)
	Kidney disease	3.2 (2.1–4.7)	1.5 (0.4–3.7)	1.017 (0.887–1.166)	1.015 (0.881–1.170)
	Depression	3.0 (1.9–4.4)	1.1 (0.2–3.2)	1.019 (0.888–1.168)	1.020 (0.885–1.176)
	Blindness	2.4 (1.4–3.7)	1.5 (0.4–3.7)	1.009 (0.880–1.157)	1.010 (0.876–1.163)
	Heart attack	1.6 (0.9–2.8)	1.9 (0.6–4.3)	0.998 (0.870–1.144)	0.998 (0.866–1.151)
	Chronic bronchitis	1.5 (0.8–2.6)	0.7 (0.1–2.7)	1.007 (0.878–1.156)	1.004 (0.871–1.158)
	Birth Defects	1.4 (0.7–2.4)	3.0 (1.3–5.8)	0.985 (0.859–1.128)	0.984 (0.855–1.134)
	High blood pressure	0.2 (0.0–0.09)	1.9 (0.6–4.3)	0.984 (0.858–1.129)	0.983 (0.853–1.133)

^*^Multiple choices were possible and the percentages (%) indicate proportions of spontaneous quotations by the subjects;^†^Adjusted for sex, age, marital status, family size, income level, and education level; ^R^Reference category; ^*^P < 0.05, ^**^P < 0.01; ^***^P < 0.001; PR, Prevalence Ratio; “yes” proportion of applicator and residents.

Perceived health risk of pesticide use [APR = 1.079 (1.004–1.159), *p* < 0.05], positive attitude to PPE use in reducing the health risk of pesticide exposure [APR = 1.081 (1.005–1.162), *p* < 0.05], but also being comfortable with current pesticide spraying practice and perceiving no own risk of pesticide poisoning [APR = 1.109 (1.005–1.223), *p* < 0.05] were significantly more frequently reported by applicators (Table 4).

**Table 4:** Attitude to pesticides among pesticide applicators and residents in East Hararge Zone, Oromiya Region, Ethiopia.

**Respondents' attitude to pesticide exposure**	**Mean**^**a**^ **(95% Cl)**		**Unadjusted PR (95% Cl)**	**Adjusted PR^†^ (95% Cl)**
	**Applicators (*n* = 803)**	**Residents^R^ (*n* = 270)**		
Effectiveness of the pesticide management system	2.7 (2.58–2.73)	2.7 (2.55–2.79)	0.991 (0.911–1.079)	0.994 (0.911–1.085)
Perceived health risk of pesticide use	4.2 (4.14–4.28)	3.9 (3.79–4.01)	1.080 (1.008–1.157)*	1.079 (1.004–1.159)*
Pesticide residues are likely to be present in the food we eat, air we breathe, water we drink and soil in the environment	4.5 (4.47–4.58)	4.5 (4.38–4.57)	1.011 (0.947–1.079)	1.011 (0.945–1.081)
Use of PPEs, such as gloves, foot, and eye protection, respirators and full body suits, reduces health risk of pesticide exposure	4.1 (4.04–4.17)	3.8 (3.67–3.89)	1.086 (1.013–1.165)*	1.081 (1.005–1.162)*
Attitude about the training of health effects of pesticides in reducing the health risk	4.5 (4.46–4.55)	4.5 (4.40–4.56)	1.005 (0.942–1.073)	1.006 (0.940–1.076)
Spraying pesticides is an ancestral practice passed down through generations and does not bring any health problems	1.4 (1.38–1.48)	1.4 (1.35–1.53)	0.991 (0.883–1.111)	0.991 (0.879–1.116)
Exposure to pesticides can induce life-threatening conditions	4.1 (4.07–4.14)	4.0 (3.99–4.02)	1.025 (0.957–1.097)	1.020 (0.950–1.096)
Comfortable with the current pesticide spraying practice and has no risk of pesticide poisoning	2.3 (2.22–2.38)	2.1 (1.93–2.20)	1.114 (1.014–1.225)*	1.109 (1.005–1.223)*

^a^Mean score of the attitude to pesticide exposure on a 5-item Likert scale (from 1 = strongly disagree to 5 = strongly agree);^†^Adjusted for sex, age, marital status, family size, income level, and education level; ^R^Reference category; ^*^P < 0.05; PR, Prevalence Ratio.

### Applied pesticides and other exposing chemicals

Based on WHO classification of pesticides by hazardousness (LD_50_), 59, 35, and 6% of reported pesticides were moderately hazardous (WHO class II), slightly hazardous (WHO class III), and unlikely to present acute hazard in normal use (WHO class IV), respectively. Insecticides (53%), herbicides (23.5%), and fungicides (23.5%) were the pesticides most frequently exposing applicators and residents in the study area. Based on the International Agency for Research on Cancer (IARC) classification, 25, 19, and 56% of reported pesticide products are probably carcinogenic to human (Group 2A), possibly carcinogenic to human (Group 2B) and not classifiable as to its carcinogenicity to human (Group 3I), respectively. Applicators were most frequently exposed to Glyphosate (40%), Malathion (35%), and Mancozeb (2.5%), while residents are the most likely to encounter Malathion (19%), Endosulfan (16%), and Diazinon (16%) (Table 5). In the present study, 77% of participants were exposed to harmful chemicals, including pesticides, at work or at home. Hundred percentage of applicators were exposed to harmful chemicals compared to 8.5% of residents. Besides pesticides, small proportions of applicators and residents were exposed to benzene (0.9 and 5.6%), kerosene (1.1 and 2.6%), diesel fuel (0.5 and 1.9%), and gasoline (0.2 and 1.5%), respectively, during use of detergents, domestic heaters and transportation at work or in home settings.

**Table 5:** Types of pesticides exposing applicators and residents in East Hararge Zone, Oromiya Region, Ethiopia.

**Pesticide type per**	**Active**	**WHO toxicity**	**IARC (EPA)**	**ADI^a^**	**Applicators**	**Residents**
**chemical group**	**ingredient**	**class**	**classification**	**(mg kg^−1^ BW day^−1^)**		
					N*(%)	N*(%)
**Herbicide**						
Glycine derivative	Glyphosate	III	2A	0.1	317 (39.5)	41 (15.2)
Phenoxy-carboxylic acid	2–4 D	II	2B	0.05	200 (24.9)	36 (13.3)
Triazine	Atrazine	III	G3	0.02	4 (0.5)	3 (1.1)
Chloroacetamide	Alachlor	III	GIII (EPA)	0.01	2 (0.2)	3 (1.1)
**Insecticide**						
Organophosphate	Malathion	III	2A	0.03	282 (35.1)	52 (19.3)
Organophosphate	Diazinon	II	2A	0.0002	253 (31.5)	43 (15.9)
Organophosphate	Dimethoate	II	G3	0.001	237 (29.5)	1 (0.4)
Pyrethroids	Deltamethrin	II	G3	0.01	135 (16.8)	43 (15.9)
Organochlorine	Endosulfan	II	G3	0.006	86 (10.7)	44 (16.3)
Organochlorine	DDT	II	2A	0.01	83 (10.3)	11 (4.1)
Carbamate	Carbaryl	II	G3	0.0075	66 (8.2)	1 (0.4)
Pyrethroids	Cypermethrin	II	2B	0.05	7 (0.9)	3 (1.1)
Organophosphate	Fenitrothion	II	G3	0.005	5 (0.6)	3 (1.1)
**Fungicide**						
Dithiocarbamate	Mancozeb	IV	G3	0.05	20 (2.5)	1 (0.4)
Chloronitrile	Chlorothalonil	III	2B	0.015	14 (1.7)	3 (1.1)
Triazole	Epoxiconazole	II	G3	0.008	4 (0.5)	3 (1.1)
Carbamic acid	Thiram	III	G3	0.01	3 (0.4)	2 (0.7)

WHO classification of pesticide by hazardousness (LD_50_): Ia = Extremely hazardous; Ib = Highly hazardous; II = Moderately hazardous; III = Slightly hazardous; IV = Unlikely to present acute hazard in normal use; International Agency for Research on Cancer (IARC) classification: Group1 (G1) = carcinogenic to humans; 2A= probably carcinogenic to humans; 2B= possibly carcinogenic to humans; G3=not classifiable as to its carcinogenicity to humans; US Environmental Protection Agency (EPA) classification: G3 = likely to be a human carcinogen at high doses; ^*^Multiple choices were possible. ^a^According to Pesticide Properties Database (PPDB).

### Diagnosed medical conditions and experienced symptoms

Asthma (12.3, 11.5%), diabetes (4.6, 10.4%), and high blood pressure (10.3, 15.2%) were diagnosed medical conditions among applicators and residents in the study areas, respectively. Prescribed drug use in the past 12 months was significantly more frequent among applicators [APR = 1.140 (1.003–1.295), *p* < 0.05]. Significantly more applicators (36%) experienced health effects presumably related to pesticide exposure than residents (15%) [APR = 1.153 (1.007–1.320), *p* < 0.05] and they also reported pesticide exposure-affected family members more frequently (31 vs. 26%, respectively), although without statistical significance (Table 6). Skin irritation [APR = 1.110 (1.005–1.225), *p* < 0.05], shortness of breath [APR = 1.092 (1.002–1.191), *p* < 0.05], cough [APR = 1.093 (1.005–1.190), *p* < 0.05], and dizziness [APR = 1.093 (1.003–1.192), *p* < 0.05] were significantly more likely reported by applicators than by their counterparts. Chest pain [APR = 0.910 (0.838–0.988), *p* < 0.05], nausea and vomiting [APR = 0.900 (0.830–0.975), *p* < 0.05], and muscle cramps [APR = 0.900 (0.830–0.975), *p* < 0.05] were more frequently reported by residents (Table 7).

**Table 6:** Existing medical conditions and health effects presumably caused by pesticide exposure among applicators and residents in East Hararge Zone, Oromiya Region, Ethiopia.

**Medical conditions**	**Applicators (*N* = 803)**	**Residents^R^ (*N* = 270)**	**Unadjusted PR (95% Cl)**	**Adjusted PR^†^ (95% Cl)**
	**Yes% (95% Cl)**	**Yes% (95% Cl)**		
Asthma	12.3 (10.1–14.8)	11.5 (7.9–15.9)	1.008 (0.884–1.148)	1.002 (0.870–1.154)
Diabetes	4.6 (3.3–6.3)	10.4 (7.0–14.6)	0.948 (0.830–1.082)	0.958 (0.830–1.106)
High blood pressure (hypertension)	10.3 (8.3–12.7)	15.2 (11.1–20.0)	0.958 (0.842–1.090)	0.963 (0.837–1.108)
Kidney and liver disease	0.0	0.0	NA	NA
Cancer	0.0	0.0	NA	NA
A prescription drug used in the past 12 months	51.1 (47.5–54.6)	31.9 (26.3–37.8)	1.146 (1.018–1.289)*	1.140 (1.003–1.295)*
Experienced health effects presumably related to pesticide exposure	36.0 (32.7–39.4)	15.9 (11.8–20.8)	1.173 (1.035–1.330)*	1.153 (1.007–1.320)*
Experienced health effects presumably related to pesticide exposure among family members	31.1 (27.9–34.5)	25.9 (20.8–31.6)	1.041 (0.922–1.177)	1.028 (0.900–1.173)

^†^Adjusted for sex, age, marital status, family size, income level, education level, past and present smoking status, frequency of alcohol consumption, and Khat consumptions; ^R^Reference category; ^*^P < 0.05; PR, Prevalence Ratio; “Yes” proportion of the applicators and residents; NA, Not applicable.

**Table 7:** Experienced symptoms among applicators and residents in East Hararge Zone, Oromiya Region, Ethiopia.

**Experienced symptoms**	**Mean**^**a**^ **(95% Cl)**		**Unadjusted PR (95% Cl)**	**Adjusted PR^†^ (95% Cl)**
	**Applicators (*n* = 803)**	**Residents^R^ (*n* = 270)**		
Skin irritation	2.5 (2.41–2.53)	2.2 (2.12–2.31)	1.116 (1.019–1.223)**	1.110 (1.005–1.225)*
Skin rashes	3.0 (2.92–3.02)	2.9 (2.81–3.01)	1.022 (0.942–1.107)	1.041 (0.954–1.136)
Eye irritation	2.6 (2.53–2.65)	2.6 (2.44–2.66)	1.016 (0.932–1.108)	1.006 (0.917–1.105)
Blurred vision	3.0 (2.94–3.05)	3.1 (2.98–3.17)	0.975 (0.901–1.055)	0.983 (0.903–1.071)
Chest pain	3.1 (3.07–3.19)	3.4 (3.30–3.50)	0.920 (0.853–0.992)*	0.910 (0.838–0.988)*
Shortness of breath	3.2 (3.09–3.22)	3.0 (2.84–3.07)	1.069 (0.987–1.157)	1.092 (1.002–1.191)*
Cough	3.3 (3.24–3.36)	3.1 (2.95–3.15)	1.083 (1.001–1.171)*	1.093 (1.005–1.190)*
Abdominal pain	3.1 (2.99–3.14)	3.0 (2.86–3.10)	1.029 (0.950–1.115)	1.042 (0.956–1.136)
Nausea and vomiting	3.1 (3.07–3.20)	3.6 (3.44–3.68)	0.880 (0.817–0.948)	0.900 (0.830–0.975)*
Diarrhea	3.1 (3.02–3.17)	3.2 (3.08–3.33)	0.965 (0.893–1.043)	0.963 (0.885–1.047)
Poor appetite	2.4 (2.29- 2.45)	2.3 (2.18–2.44)	1.028 (0.939–1.125)	1.025 (0.930–1.131)
Fatigue	1.8 (1.75–1.89)	1.8 (1.67–1.92)	1.013 (0.914–1.122)	1.013 (0.906–1.132)
Difficulty to concentrate	3.0 (2.92–3.03)	2.9 (2.74–2.96)	1.043 (0.962–1.131)	1.038 (0.950–1.133)
Forgetfulness	2.9 (2.85–2.99)	2.9 (2.80–3.05)	0.999 (0.921–1.082)	1.004 (0.920–1.095)
Dizziness	3.2 (3.10–3.25)	2.9 (2.81–3.05)	1.083 (1.000–1.173)*	1.093 (1.003–1.192)*
Headache	3.1 (3.04–3.18)	3.0 (2.90–3.09)	1.037 (0.958–1.123)	1.052 (0.965–1.146)
Muscle cramps	3.5 (3.43–3.57)	3.9 (3.73–3.96)	0.910 (0.847–0.977)**	0.897 (0.830–0.969)*
Numbness in the arms or legs	3.3 (3.27–3.41)	3.5 (3.37–3.60)	0.958 (0.890–1.032)	0.955 (0.881–1.035)

^a^Mean score of the frequency of experienced symptoms measured by 5-item Likert scale (from 1= always to 5= never);^†^Adjusted for sex, age, marital status, family size, income level, education level, past and present smoking status, frequency of alcohol consumption, and Khat consumptions; ^R^Reference category; ^*^P < 0.05, ^**^P < 0.01; PR, Prevalence Ratio.

### Pesticide use and disposal

In this study, all applicators used manual backpack sprayers, and none of them had license for pesticide application. Sixty-nine percent and 80% of the respondents reported that leftover pesticide residues were sold/offered to other fellow farmers and disposed empty pesticide containers on an open field, respectively (Table 8). A considerable proportion of the respondents followed risky behaviors, including chewing Khat, smoking cigarette, and drinking water or eating food during spraying pesticides (Table 8).

**Table 8:** Hazardous practices of applicators during spraying and disposal of pesticides in East Hararge Zone, Oromiya Region, Ethiopia.

**Characteristics (*n* = 803)**	**Options**	**Applicators**
		N*(%)
Practices during spraying pesticides	Chew Khat	447 (55.7)
	Smoke cigarette	361 (45.0)
	Drink water, eat food	281 (35.0)
	Do nothing	280 (34.9)
Pesticide residue disposal system	Sell/offer to other farmers	554 (69.0)
	Buy only the amount used	348 (43.3)
	Dump on an open field	256 (31.9)
	Burry	255 (31.8)
Disposal of empty pesticide containers	Dump on an open field	644 (80.2)
	Use for storage of other pesticides	476 (59.3)
	Burry	355 (44.2)
	Burn	283 (35.2)

^*^Multiple choices were possible.

Perceived toxicity of currently applied pesticide products [APR = 1.404 (1.234–1.598), *p* < 0.001] mixing pesticides with a stick without wearing gloves [APR = 1.129 (1.002–1.272), *p* < 0.05], washing spray tank after application [APR = 1.286 (1.130–1.463), *p* < 0.001], occurrence of an incidental splash during mixing and application [APR = 1.139 (1.008–1.287), *p* < 0.05], and regular maintenance of sprayer tank [APR = 1.299 (1.142–1.478), *p* < 0.001] were significantly associated with health effects among applicators (Table 9). In the total study population, health effects were 16% more frequently experienced among those who reported exposure to harmful chemicals at work or at home [CPR = 1.165 (1.023–1.326), *p* < 0.05], but the association disappeared after adjustment for potential confounders.

**Table 9:** Association between pesticide use and exposure, and health effects among pesticide applicators in East Hararge Zone, Oromiya Region, Ethiopia.

**Pesticide use and exposure (*n* = 803)**	**Options**	**%**	**Experienced health effects**			
			**No (*n* = 514)**	**Yes (*n* = 289)**	**Unadjusted PR (95% Cl)**	**Adjusted PR (95% Cl)^†^**
Toxicity of pesticide products currently applied	Highly toxic	26.5	12.3%	51.9%	1.379 (1.216–1.564)***	1.404 (1.234–1.598)***
	Slightly toxic	73.5	87.7%	48.1%	1.00	1.00
Years of pesticide application	1–5 years	20.8	19.3%	23.5%	1.102 (0.913–1.330)	1.101 (0.912–1.331)
	5–9 years	59.4	58.4%	61.2%	1.074 (0.917–1.257)	1.073 (0.915–1.258)
	over 10 years	19.8	22.4%	15.2%	1.00	1.00
Length of a single application (minutes)	46–65	54.2	49.6%	62.3%	1.091 (0.968–1.229)	1.091 (0.966–1.233)
	25–45	45.8	50.4%	37.7%	1.00	1.00
Frequency of pesticide application in a month	Two sessions	52.4	49.0%	58.5%	1.066 (0.947–1.201)	1.068 (0.946–1.206)
	One session	47.6	51.0%	41.5%	1.00	1.00
Trend of pesticide use	Increasing	45.5	42.8%	50.2%	1.052 (0.934–1.184)	1.059 (0.940–1.194)
	Decreasing	54.5	57.2%	49.8%	1.00	1.00
Place of storing pesticides before application	In the house	17.8	17.7%	18.0%	1.093 (0.902–1.325)	1.102 (0.908–1.337)
	In the house yard	60.0	56.2%	66.8%	1.123 (0.965–1.307)	1.128 (0.969–1.314)
	In secured warehouse	22.2	26.1%	15.2%	1.00	1.00
Method of mixing pesticides	With a stick without gloves	48.8	42.6%	59.9%	1.124 (0.998–1.266)	1.129 (1.002–1.272)*
	With a stick wearing gloves	51.2	57.4%	40.1%	1.00	1.00
Washing the sprayer tank after application	Yes	61.1	49.0%	82.7%	1.281 (1.130–1.453)***	1.286 (1.130–1.463)***
	No	38.9	51.0%	17.3%	1.00	1.00
Regular maintenance of the sprayer tank	Yes	59.4	47.1%	81.3%	1.281 (1.131–1.450)***	1.299 (1.142–1.478)***
	No	40.6	52.9%	18.7%	1.00	1.00
Incident of pesticide splash during mixing, application, and tank wash	Yes	55.7	48.8%	67.8%	1.141 (1.011–1.287)*	1.139 (1.008–1.287)*
	No	44.3	51.2%	32.2%	1.00	1.00

^†^Adjusted for sex, age, marital status, family size, income level, and education level, past and present smoking status, frequency of alcohol consumption, and Khat consumptions; ^*^P < 0.05, ^***^P < 0.001, PR, Prevalence Ratio.

### Preventive measures

Ninety-five percent (764) and 76% (610) of the respondents acquired information about the health risks of pesticide exposure from agricultural extension workers and health extension workers, respectively. Fifty-eight percent of the applicators attended training on the health risks of pesticides, pesticide use, management, and application, and 50% of them followed the label instructions found on pesticide containers. Fifty-four percent and 53% of the respondents reported changing their clothes after the application and taking a shower immediately after spraying, respectively. Being very expensive (53%) or unavailability in the local market (47%) were the main reasons for not or rarely using preventive measures in this study population. All the applicators reported that they use safety glasses (goggles) sometimes. Use of face mask [APR = 1.119 (1.002–1.250), *p* < 0.05] and visiting health facility when experiencing a symptom presumably due to pesticide exposure [APR = 1.194 (1.060–1.345), *p* < 0.01) were significantly positively correlated with attending training on the health risks and use of pesticides (Table 10). Use of respirators [APR = 0.705 (0.615–0.807), *p* < 0.001], gloves [APR = 0.892 (0.798–0.996), *p* < 0.05], safety shoes [APR = 0.764 (0.674–0.867), *p* < 0.001], and use of home-based care after experiencing a symptom presumably due to pesticide exposure [APR = 0.889 (0.792–0.996), *p* < 0.05] showed a significant negative correlation with training (Table 10).

**Table 10:** Association between completing training on the health risks and use of pesticides, and applying preventive measures among pesticide applicators in East Hararge Zone, Oromiya Region, Ethiopia.

**Preventive measures (*n* = 803)**	**Options**	**%**	**Attended training on the health risks and use of pesticides**			
			**No (*n* = 338)**	**Yes (*n* = 465)**	**Unadjusted PR (95% Cl)**	**Adjusted PR (95% Cl)†**
Read and follow the label instructions on pesticide containers	Yes	50.1	43.2%	55.1%	1.076 (0.902–1.284)	1.078 (0.964–1.205)
	No	49.9	56.8%	44.9%	1.00	1.00
Use face mask	Always	46.7	38.8%	52.5%	1.119 (1.002–1.250)	1.119 (1.002–1.250)*
	Sometimes	53.3	61.2%	47.5%	1.00	1.00
Use respirator	Always	28.0	52.4%	10.3%	0.705 (0.575–0.864)***	0.705 (0.615–0.807)***
	Sometimes	72.0	47.6%	89.7%	1.00	1.00
Use gloves	Always	48.4	58.6%	41.1%	0.897 (0.752–1.071)	0.892 (0.798–0.996)*
	Sometimes	51.6	41.4%	58.9%	1.00	1.00
Use rubber boots	Always	32.0	52.7%	17.0%	0.766 (0.631–0.930)**	0.764 (0.674–0.867)***
	Sometimes	68.0	47.3%	83.0%	1.00	1.00
Use coveralls	Always	47.7	50.9%	45.4%	0.966 (0.810–1.154)	0.961 (0.859–1.076)
	Sometimes	52.3	49.1%	54.6%	1.00	1.00
Reasons for not using or rarely using preventive measures	Very expensive	47.9	42.3%	52.0%	1.062 (0.890–1.267)	1.058 (0.947–1.184)
	Unavailable in local marker	52.1	57.7%	48.0%	1.00	1.00
Changing clothes after application	Yes	53.8	59.2%	49.9%	0.948 (0.794–1.132)	0.948 (0.849–1.059)
	No	46.2	40.8%	50.1%	1.00	1.00
Taking shower immediately after spraying	Always	52.7	57.4%	49.2%	0.951 (0.797–1.135)	0.953 (0.852–1.065)
	Sometimes	47.3	42.6%	50.8%	1.00	1.00
Visited health facility when experienced a symptom presumably due to pesticide exposure	Yes	63.8	48.2%	75.1%	1.202 (1.069–1.353)**	1.194 (1.060–1.345)**
	No	36.2	51.8%	24.9%	1.00	1.00
Used home-based care when experienced a symptom presumably due to pesticide exposure	Yes	42.1	53.3%	34.0%	0.922 (0.771–1.102)	0.889 (0.792–0.996)*
	No	57.9	46.7%	66.0%	1.00	1.00

^†^Adjusted for sex, age, marital status, family size, income level, and education level, past and present smoking status, frequency of alcohol consumption, and Khat consumptions; ^*^P < 0.05, ^**^P < 0.01, ^***^P < 0.001; PR, Prevalence Ratio.

Experiencing health effects was significantly positively correlated with using home-based care [APR = 1.130 (1.002–1.276), *p* < 0.05]; using face mask, not using PPE because expensive, and visiting health facilities were also positively correlated but without statistical significance (Table 11).

**Table 11:** Association between experienced health effects and applying preventive measures among pesticide applicators in East Hararge Zone, Oromiya Region, Ethiopia.

**Preventive measures (*n* = 803)**	**Options**	**%**	**Experienced health effects**			
			**No (*n* = 514)**	**Yes (*n* = 289)**	**Unadjusted PR (95% Cl)**	**Adjusted PR (95% Cl)^†^**
Read and follow the label instructions on pesticide containers	Yes	50.1	50.4%	49.5%	0.994 (0.883–1.119)	0.992 (0.880–1.119)
	No	49.9	49.6%	50.5%	1.00	1.00
Use face mask	Always	46.7	44.7%	50.2%	1.036 (0.920–1.167)	1.034 (0.917–1.164)
	Sometimes	53.3	55.3%	49.8%	1.00	1.00
Use respirator	Always	28.0	30.0%	24.6%	0.955 (0.836–1.092)	0.953 (0.833–1.092)
	Sometimes	72.0	70.0%	75.4%	1.00	1.00
Use gloves	Always	48.4	50.4%	45.0%	0.964 (0.856–1.086)	0.963 (0.855–1.085)
	Sometimes	51.6	49.6%	55.0%	1.00	1.00
Use rubber boots	Always	32.0	34.4%	27.7%	0.948 (0.834–1.078)	0.947 (0.832–1.079)
	Sometimes	68.0	65.6%	72.3%	1.00	1.00
Use coveralls	Always	47.7	50.2%	43.3%	0.954 (0.847–1.074)	0.958 (0.849–1.082)
	Sometimes	52.3	49.8%	56.7%	1.00	1.00
Reasons for not using or rarely using preventive measures	Very expensive	47.9	46.7%	50.2%	1.024 (0.909–1.153)	1.024 (0.908–1.155)
	Unavailable in local marker	52.1	53.3%	49.8%	1.00	1.00
Changing clothes after application	Yes	53.9	57.0%	48.4%	0.943 (0.838–1.062)	0.943 (0.837–1.062)
	No	46.1	43.0%	51.6%	1.00	1.00
Taking shower immediately after spraying	Always	52.7	54.3%	49.8%	0.970 (0.862–1.093)	0.970 (0.861–1.094)
	Sometimes	47.3	45.7%	50.2%	1.00	1.00
Visited health facility when experienced a symptom presumably due to pesticide exposure	Yes	63.8	61.3%	68.2%	1.052 (0.929–1.191)	1.053 (0.928–1.193)
	No	36.2	38.7%	31.8%	1.00	1.00
Used home-based care when experienced a symptom presumably due to pesticide exposure	Yes	42.1	35.6%	53.6%	1.132 (1.005–1.276)*	1.130 (1.002–1.276)*
	No	57.9	64.4%	46.4%	1.00	1.00

^†^Adjusted for sex, age, marital status, family size, income level, and education level, past and present smoking status, frequency of alcohol consumption, and Khat consumptions; ^*^P < 0.05, PR, Prevalence Ratio.

## Discussion

This study investigates occupational pesticide exposure, related health risks and associated factors among pesticide applicators compared to residents in Ethiopia. Numerous risky behaviors related to occupational pesticide exposure were identified, which placed the applicators to increased health risks. Based on the current findings, noticeable variations were observed in all aspects of knowledge of and attitude toward pesticide use and exposure, experienced medical conditions and symptoms between pesticide applicators and residents.

High level of knowledge about pesticides is indispensable for applicators to use comprehensive strategies to reduce human health risks. Applicators in this study most frequently indicated that inhalation, water contamination, and asthma were the most important route of exposure, the major problem of the environment associated with pesticide use, and the most frequently reported health effect pesticide exposure can induce, respectively. Regular training and raising awareness of applicators about pesticide routes of exposure are essential since uptake through dermal exposure can also be high in occupational settings (46). The overall knowledge about pesticide hazards was significantly higher among applicators than residents which may be attributed to the fact that applicators more regularly deal with pesticides and have a higher opportunity to get training from different sources than residents. Our study revealed a higher level of knowledge than a previous study by Mequanint et al. (47), Mergia et al. (48), and Endalew et al. (49) but led to similar findings as investigations done in Ethiopia by Mengistie et al. (50) and Gesesew et al. (42), and in Tanzania by Lekei et al. (43). However, having good knowledge of pesticide hazards doesn't necessarily translate into best practices of pesticide handling and preventive measures. Hence, continuous monitoring and evaluation of application practice during field spraying are crucial because a substantial proportion of study participants have a low level of education and usually well-materialize their knowledge into practices and adopt protective behaviors through learning by doing in the field.

Misunderstanding concerning pesticides can impair the aptitude of applicators to adopt self-protective behaviors against the health risks of work-related pesticide exposure and to prevent short and long-term health effects. In this study, applicators reported a statistically significantly higher mean score on many attributes of attitude than residents. For instance, attitude related to the perceived health risks of pesticide use and their consequences, and use of PPEs in reducing the health risk of pesticide exposure showed a significantly higher mean score among applicators. A similar perception was reported in Brazil by Pasiani et al. (51) and Recena et al. (52) and by Hamid in Malaysia (53). In addition, a substantial percentage of applicators thought that exposure to pesticides might induce life-threatening conditions. However, a substantial proportion of them reported that they are comfortable with the existing spraying practice. This inconsistency in perception gave an indication that the applicators did not show a readiness or willingness to change their present pesticide use practices. Evidence-based interactive education, practical-oriented, target-specific behavioral change, risk communication strategies regarding pesticide use and preventive measures are necessary. The gap between the knowledge, attitude and actual safety practices need to be linked with a more multifaceted and participatory training model and behavioral interventions.

Insecticide, herbicide, and fungicide products were reported to be used frequently by applicators at a ratio of 9:4:4. Based on the WHO hazard classification of pesticides, 59 and 35% of reported pesticide products are moderately and slightly hazardous, respectively (54). Based on the International Agency for Research on Cancer (IARC) classification, 25 and 19% of reported pesticide products are probably (Group 2A, e.g., Glyphosate, Malathion, Diazinon, and DDT) and possibly (Group 2B, e.g., 2,4-D, Cypermethrine, and Chlorothalonil) carcinogenic to humans, respectively. In addition to acute health risks, some reported pesticides might tend to be persistent in the environment and bioaccumulate in the food chain (e.g., Deltamethrin, DDT, Endosulfan, and Cypermethrine) and pose chronic health risks (55–57). Use of the toxic and persistent organochlorine pesticides, such as DDT and Endosulfan that had already been banned in most countries worldwide were reported from the study area (58, 59). In Ethiopia, DDT is still actively sprayed for malaria vector control by the Ministry of Health; however, applicators also apply it to food crops and Khat (Catha edulis) in illegal ways (60, 61). Many illegal pesticides are still used widely in Ethiopia due to the inefficient pesticide management system. Ineffective extension services have been considered as critical factors leading to the misuse of pesticides, as indicated in our previous study (35).

A significant proportion of applicators reported that they experienced health effects presumably related to pesticide exposure at some point in the past, similar to what was reported among Bolivian (62) and Thai farmers (63). Moreover, almost one-third of them also reported that they encountered health effects among their family members probably related to pesticide poisoning. Para-occupational or take-home exposure pathways are potential sources of exposure for this population. Chemicals used in agriculture, including pesticides, can move from the workplace to residential environments and eventually lead to elevated concentration of pesticide residues over time, particularly in house dust and vehicle dust. It is also possible that applicators brought pesticides home for residential use, and the inappropriate handling and storage of these products constitute potential health risk for those living in the household, especially for children (64, 65). Although significantly more applicators used prescription drugs in the past 12 months, diabetes and high blood pressure were less reported among applicators compared to residents, which may typically be a result of “healthy worker effect” as observational studies are particularly prone to this type of bias (66). A range of adverse symptoms that were related to pesticide exposure reported by other studies (67), such as skin irritation, shortness of breath, cough, and dizziness have also been more frequently reported by applicators then residents in this study. On the other hand, chest pain, nausea and vomiting, and muscle cramps were significantly less likely reported by applicators. This may be due to the “healthy worker effect” (66), or to other confounding factors that have not been assessed in this study. Similar findings which also support the present observation were reported from Costa Rica where the loss of appetite, paleness, stomach pain and nausea were more frequently observed in farmers who never experienced health effects than in those who experienced at least one following pesticide application (68).

The effective functioning of agricultural and health extension workers plays an integral role in providing information and guidance on proper pesticide use, providing technical services and improving farmers' awareness and behaviors (35). The extension workers are the main channels for disseminating information about pesticide use to the applicators, although the degree of support may be inadequate (35, 69, 70). Storage and disposal of leftover pesticide residues and empty containers are critical points of intervention to enhance safety awareness before, during, and after the application of pesticides. In the present study, a higher proportion (69%) of applicators sold or offered leftover pesticide residue to other farmers; however, a considerable proportion (43%) of applicators purchase only the amount of pesticide that is needed for the application, which is the best practice that should be encouraged. On the other hand, a high proportion (80%) of applicators disposed empty pesticide containers on open fields, whereas 59% of them also used empty containers to store other pesticides. Similar figures are reported in other studies (71, 72). This practice may not only subject the applicators to a high risk of pesticide exposure, but also endanger the health of family members, residents, and bystanders through non-occupational exposure pathways. Hence, the appropriate collection, recycling and disposal of empty pesticide containers should be implemented to reduce human health risk and environmental pollution.

A significant proportion of the applicators practiced risky behaviors during pesticides application. They either chew Khat, smoke cigarettes, drink or eat during spraying pesticides. Similar findings were reported from Gaza by Yassin et al. (72). A substantial proportion of them used home-based care treatment when experiencing symptoms presumably due to pesticide exposure. This is probably because of financial constraints, low level of education and low health literacy. This practice could worsen the illness and lead to severe consequences due to the missed opportunity of early detection and adequate treatment (73), which also result in higher healthcare costs (74).

Our findings showed that perceiving pesticides as highly toxic, mixing pesticides without gloves, regular maintenance and washing spray tank after application, and incidents of splash during mixing and application were significantly positively associated with experienced health effect presumably related to pesticide exposure among applicators. Similar findings were observed in Rwanda (75). A considerable proportion of applicators rated the toxicity of applied pesticide products as slightly toxic, although several of them can express high toxicity together with their bioaccumulation tendency and synergistic effects, which may undermine the adequate use of preventive measures. Similar findings are reported by Memon et al. (74) and Jallow et al. (76).

The use of protective measures among applicators is an indispensable factor for the reduction of health risks of occupational pesticide exposure. The cost of preventive measures is considerably lower than the cost of medical treatment of health effects from exposure to pesticides (74); hence, preventive measures are not only more ethical but also more cost-effective and feasible strategies to combat these issues, especially in low- and middle-income countries, including Ethiopia. In the present study, about half of the applicators did not read and follow the label instructions on the pesticide containers, and only less than half of them used always face mask during spraying, although significantly more among those who received tailored training. About the same proportion used gloves and coveralls, although, interestingly, self-reported use of gloves was more frequent among non-trained applicators. The use of respirators and safety shoes was mainly intermittent. A similar conclusion was drawn by Ndayambaje et al. (75) in Rwanda and Orozco et al. (77) in Ecuador. Training about effective and consistent utilization of PPEs is crucial. Availability of PPEs at an affordable cost in the local market is also a key factor for the adequate use of preventive measures. Changing cloth after application and taking a shower immediately after application is critical in reducing the health risk of occupational pesticide exposure among applicators (78, 79). It additionally reduces risk to family members that may face take-home exposure pathways. This study also showed that applicators who experienced health effects significantly more frequently used home-based care, but not health facilities. Therefore, comprehensive training focusing on basic safety precautions, proper choice and use of PPEs, and visiting health facilities at onset of potentially pesticide-related symptoms are crucial interventions for reducing the risk of developing pesticide-related health effects in the occupational setting.

## Strength and limitations

As the study used a cross-sectional design, a temporal causal relationship between exposure and outcome could not be determined because both were examined at the same time. The collected information was self-reported; therefore, it may be subject to recall and social desirability bias. In addition to self-reported exposure status, exposure levels were not confirmed by environmental or biological monitoring, but we can assume that pesticide exposure levels are higher in the occupational than in the residential settings. Although the presence of confounders cannot be excluded, probable absence of strong confounding was confirmed by the lack of significant variations (< 10%) between crude and adjusted PRs in most cases. In the effort made to address these limitations, a questionnaire-based survey of face-to-face interviewer-administered interviews was conducted on a large random sample of pesticide applicators and residents providing a high degree of reliability and representativeness for the target working population as well as for the general public.

## Conclusions

In the present study, circumstances and key determinants of the health risks of occupational exposure to pesticides were identified. Enhancing formal education of the applicators and employment training programs for strict utilization of adequate collective and personal protective measures are essential to further reduce health risks. Enhancing the general knowledge of pesticide hazards, correcting erroneous perception, avoiding risky behaviors during pesticide use, pursuing safe practice of pesticide application, handling, storage, and disposal, ensuring the access to appropriate PPEs and other preventive measures at the local market with affordable cost and the continuous monitoring and evaluation of these activities are crucial for the better protection of pesticide users and the general public. Finally, further investigation of pesticide exposure by directly evaluating the actual occupational health risks of pesticide applicators using environmental and biological monitoring is recommended.

## Data availability statement

The raw data supporting the conclusions of this article will be made available by the authors, without undue reservation.

## Ethics statement

The studies involving human participants were reviewed and approved by Institutional Health Research Ethics Review Committee (IHRERC), College of Health and Medical Science, Haramaya University, Ethiopia. The patients/participants provided their written informed consent to participate in this study.

## Author contributions

BÁ: conceptualization. RT and BÁ: design of the work. RT: acquisition, analysis, interpretation of the data, and writing—original draft preparation. BÁ and KN: interpretation of the data and writing review and editing. All authors approved the final version of the manuscript to be published and agree to be accountable for all aspects of the work.

## Funding

The work was supported by Tempus Public Foundation under the Stipendium Hungaricum Scholarship Programme for RT.

## Conflict of interest

The authors declare that the research was conducted in the absence of any commercial or financial relationships that could be construed as a potential conflict of interest.

## Publisher's note

All claims expressed in this article are solely those of the authors and do not necessarily represent those of their affiliated organizations, or those of the publisher, the editors and the reviewers. Any product that may be evaluated in this article, or claim that may be made by its manufacturer, is not guaranteed or endorsed by the publisher.
