# Supplementary material for: Occupational and environmental pesticide exposure and associated health risks among pesticide applicators and non-applicator residents in rural Ethiopia
Source: Front Public Health. 2022 Dec 1;10:1017189. doi: 10.3389/fpubh.2022.1017189 (PMC9751888; doi:10.3389/fpubh.2022.1017189)
Supplement: Supplementary file 1 [file Data_Sheet_1.pdf]

**Supplementary file:**

**Pesticide exposure survey questionnaire**

This study investigates health risk of occupational pesticide exposure among pesticide applicators and residents in east Hararge Ethiopia using a structured survey questionnaire. The questionnaire collects data on socio-economic and demographic factors, knowledge on pesticides, pesticide handling and application practice, risk perception, health effects and confounding factors.

| | |
|---|---|
| **Name of the district** | **Questionnaire ID_____ HH ID____** |
| **Name of kebele**<br>__________________ | Supervisor name and signature<br>_________________________ |
| **Day /Month /Year**<br>______/_____/______ | Completed ☐<br>Not completed ☐ |

## Part I: Socio-demographic characteristics and lifestyle

| No | Question | Options | Tick |
|---|---|---|---|
| 1 | Sex | Male<br>Female | ☐<br>☐ |
| 2 | Age in years | Specify: __________________ | |
| 3 | Marital status | Married<br>Single<br>Divorced/Separated<br>Widowed | ☐<br>☐<br>☐<br>☐ |
| 4 | How many family members live in your household? | ≤3<br>>3 | ☐<br>☐ |
| 5 | How many children less than 5 years old live in your household? | No under 5 children<br>≤ 2<br>≥3 | ☐<br>☐<br>☐ |
| 6 | What is the approximate monthly income per person in your family? | Below average: <9000 ETB<br>Above average: ≥9000 ETB | ☐<br>☐ |
| 7 | What is your educational status? | Illiterate<br>Primary (gr. 1-8)<br>Secondary (gr. 9-12)<br>Tertiary (gr. 12+) | ☐<br>☐<br>☐<br>☐ |
| 8 | Do you currently smoke tobacco on daily basis, less than daily or not at all?<br>*If the respondent says Not at all, go to Q10.* | Daily<br>Less than daily<br>Not at all | ☐<br>☐<br>☐ |
| 9 | On average, how many of the following products do you currently smoke each week?<br>*Write # of items/sessions per week for each product.* | Manufactured cigarette: __________________<br>Hand-rolled cigarette: __________________<br>Waterpipe sessions: ____________________<br>Others, specify: _______________________ | |
| 10 | In the past, have you smoked tobacco on daily basis, less than daily, or not at all? | Daily<br>Less than daily<br>Not at all | ☐<br>☐<br>☐ |
| 11 | How often do you have a drink containing alcohol?<br>*If the respondent says Never, go to Q14.* | Never<br>Monthly or less<br>2-4 times a month<br>2- 3 times a week<br>≥ 4 times a week | ☐<br>☐<br>☐<br>☐<br>☐ |

| | | | |
|---|---|---|---|
| 12 | How many drinks containing alcohol do you have on a typical day when you are drinking? | 1-2 drinks<br>3-4 drinks<br>5- 6 drinks<br>7-9 drinks<br>$\geq$ 10 drinks | ☐<br>☐<br>☐<br>☐<br>☐ |
| 13 | How often do you have six or more drinks on one occasion? | Never<br>Less than monthly<br>Monthly<br>Weekly<br>Almost daily | ☐<br>☐<br>☐<br>☐<br>☐ |
| 14 | Do you chew khat (*Catha edulis*) on daily basis, less than daily, or not at all? | Daily<br>Less than daily<br>Not at all | ☐<br>☐<br>☐ |
| | **Part II: Knowledge about pesticides** | | |
| 15 | Do you know any pesticide product by name?<br>*If the respondent says No, go to Q17.* | Yes<br>No | ☐<br>☐ |
| 16 | List as many pesticide products as you can: ______________________________ | | |
| 17 | Do you know the routes through which pesticides can enter the human body? *If the respondent says No, go to Q 19.* | Yes<br>No | ☐<br>☐ |
| 18 | Specify as many routes as you can: ______________________________ | | |
| 19 | Do you know the major problems of the environment associated with pesticide use? *If the respondent says No, go to Q 21.* | Yes<br>No | ☐<br>☐ |
| 20 | Specify as many problems as you can: ______________________________ | | |
| 21 | Do you know any health effects pesticide exposure can induce?<br>*If the respondent says No, go to Q23.* | Yes<br>No | ☐<br>☐ |
| 22 | Specify as many health effects as you can: ______________________________ | | |
| 23 | What are your sources of information related to the health risks of pesticides?<br>*Respondent can tick more than one option.* | Health extension workers<br>Agricult. extension workers<br>Model farmers<br>Mass media<br>Pesticide retailers<br>Other, specify: ____________ | ☐<br>☐<br>☐<br>☐<br>☐ |
| | **Part III: Risk perception related to pesticide exposure** | | |

| | | | | | | | |
|---|---|---|---|---|---|---|---|
| | *Respondent should rate the following statements from 1 to 5 as how much they agree with them (1= strongly disagree to 5= strongly agree).* | I don't know | 1 | 2 | 3 | 4 | 5 |
| 24 | The current pesticide distribution, storage, handling, and utilization system is effective. | ☐ | ☐ | ☐ | ☐ | ☐ | ☐ |
| 25 | The way you currently apply pesticides poses risk to your health. | ☐ | ☐ | ☐ | ☐ | ☐ | ☐ |
| 26 | Pesticide residues are likely to be present in the food we eat, air we breathe, water we drink and soil in the environment. | ☐ | ☐ | ☐ | ☐ | ☐ | ☐ |
| 27 | Use of PPEs, such as gloves, foot, and eye protection, respirators and full body suits, reduces health risk of pesticide exposure. | ☐ | ☐ | ☐ | ☐ | ☐ | ☐ |
| 28 | Training about the health effects of pesticides, their proper handling and safe application can reduce health risk of pesticide exposure. | ☐ | ☐ | ☐ | ☐ | ☐ | ☐ |
| 29 | Spraying pesticide is an ancestral practice passed down through generations, it does not bring any health problem. | ☐ | ☐ | ☐ | ☐ | ☐ | ☐ |
| 30 | Exposure to pesticides can induce life threatening conditions and shorten the life span. | ☐ | ☐ | ☐ | ☐ | ☐ | ☐ |
| 31 | I am comfortable with the current spraying practice; I have no risk of pesticide poising. | ☐ | ☐ | ☐ | ☐ | ☐ | ☐ |

## Part IV: General health

| | | | |
|---|---|---|---|
| 32 | Have you taken prescription drugs in the past 12 months?<br>*If the respondent says No, go to Q34.* | Yes<br>No | ☐<br>☐ |
| 33 | Name of drug(s): ________________________ Specify drug(s) used for: __________________________________ | | |
| 34 | Have you ever had any of the following medical conditions?<br>*If respondent says Yes, please ask to indicate the year of diagnosis.* | | |

| Medical conditions | No | Yes | Specify year of diagnosis |
|---|---|---|---|
| Asthma | ☐ | ☐ | _____________ |
| Diabetes | ☐ | ☐ | _____________ |
| Cardiovascular disease (e.g. high blood pressure, heart attack, stroke, etc.) → Specify type of disease: ______________________ | ☐ | ☐ | _____________ |
| Kidney disease → Specify type of disease: ___________________ | ☐ | ☐ | _____________ |
| Liver disease → Specify type of disease: ____________________ | ☐ | ☐ | _____________ |
| Cancer → Specify type of disease: _________________________ | ☐ | ☐ | _____________ |
| Other chronic condition → Specify disease: ___________________ | | | _____________ |

## Part V: Experienced symptoms

35 How often have you experienced the following symptoms in recent months?

| *Respondent should indicate how often in recent months they have experienced a particular symptom.* | Always | Often | Some times | Seldom | Never |
|---|---|---|---|---|---|
| Skin irritation | ☐ | ☐ | ☐ | ☐ | ☐ |
| Skin rushes | ☐ | ☐ | ☐ | ☐ | ☐ |
| Eye irritation | ☐ | ☐ | ☐ | ☐ | ☐ |
| Blurred vision | ☐ | ☐ | ☐ | ☐ | ☐ |
| Chest pain | ☐ | ☐ | ☐ | ☐ | ☐ |
| Shortness of breath | ☐ | ☐ | ☐ | ☐ | ☐ |
| Cough | ☐ | ☐ | ☐ | ☐ | ☐ |
| Abdominal pain | ☐ | ☐ | ☐ | ☐ | ☐ |
| Nausea and vomiting | ☐ | ☐ | ☐ | ☐ | ☐ |
| Diarrhea | ☐ | ☐ | ☐ | ☐ | ☐ |
| Poor appetite | ☐ | ☐ | ☐ | ☐ | ☐ |
| Fatigue | ☐ | ☐ | ☐ | ☐ | ☐ |
| Difficulty to concentrate | ☐ | ☐ | ☐ | ☐ | ☐ |
| Forgetfulness | ☐ | ☐ | ☐ | ☐ | ☐ |
| Dizziness | ☐ | ☐ | ☐ | ☐ | ☐ |
| Headache | ☐ | ☐ | ☐ | ☐ | ☐ |
| Muscle cramps | ☐ | ☐ | ☐ | ☐ | ☐ |
| Numbness in the arms or legs | ☐ | ☐ | ☐ | ☐ | ☐ |

| | | | |
|---|---|---|---|
| 36 | Have you ever experienced any health effect presumably related to pesticide exposure/poisoning? | Yes<br>No | ☐<br>☐ |
| 37 | Have you ever encountered any health consequences of pesticide exposure/poising among your family members? | Yes<br>No | ☐<br>☐ |

| | **Part VI: Pesticide use and exposure** | | |
|---|---|---|---|
| 38 | Have you been exposed to harmful chemicals in your work or at home? *If the respondent says No, go to Q 40.* | Yes<br>No | ☐<br>☐ |
| 39 | Please specify the chemical(s) and exposure period | | |
| | Specify the chemical | For how many years exposed | |
| | Pesticide → Specify: ________________________________ | ______________<br>____________ | |
| | Organic solvent → Specify: ___________________________ | ______________<br>____________ | |
| | Others → Specify: __________________________________ | ______________<br>____________ | |
| 40 | Do you currently use pesticides in your farmland?<br>*If the respondent says No, the questionnaire ends here.* ***Thank you very much for your cooperation!*** | Yes<br>No | ☐<br>☐ |
| 41 | Specify type of pesticides typically used :_____________________________________________ | | |
| 42 | How would you rate the toxicity of the pesticide product(s) currently applied? | Highly toxic<br>Slightly toxic<br>Not toxic | ☐<br>☐<br>☐ |
| 43 | Do you have a pesticide applicator license? | Yes<br>No | ☐<br>☐ |
| 44 | For how many years have you used pesticides in your farmland? | ≤1years<br>1-5 years<br>5-9 years<br>Over 10 years | ☐<br>☐<br>☐<br>☐ |
| 45 | On average, how long time does a pesticide application take? | Minutes: _______ | |
| 46 | How many times do you apply pesticides in a month? | Number: _______ | |
| 47 | In the past 5 years, have you used a decreasing, increasing or unchanged amount of pesticide products in your farmland? | Decreasing<br>Increasing<br>Constant | ☐<br>☐<br>☐ |
| 48 | Which application mode do you use to apply pesticides?<br>*Respondent can select more than one option.* | Pressurized hand sprayer<br>Manual backpack sprayer<br>Tractor operated sprayer | ☐<br>☐<br>☐ |
| 49 | How do you mix pesticides? | With bare hands<br>With a stick<br>With hands wearing gloves<br>With a stick wearing gloves | ☐<br>☐<br>☐<br>☐ |
| 50 | Do you wash the sprayer tank after the application? | Yes<br>No | ☐<br>☐ |
| 51 | Have you faced any incidents of splash or spill of pesticide droplets during mixing, application and tank wash? | Yes<br>No | ☐<br>☐ |
| 52 | Is the sprayer tank maintained regularly? | Yes<br>No | ☐<br>☐ |
| 53 | Where do you store the pesticides before application? | In secured warehouse<br>In the house yard<br>In the house | ☐<br>☐<br>☐ |
| 54 | How do you dispose off the leftover pesticide?<br>*Respondent can select more than one option.* | Burn<br>Burry<br>Dump on open field<br>Sell/offer to other farmers<br>Return to waste management site<br>Other, specify: ____________________ | ☐<br>☐<br>☐<br>☐<br>☐ |

| | | | |
|---|---|---|---|
| 55 | Where do you put the used empty containers?<br>*Respondent can select more than one option.* | Burn<br>Burry<br>Dump on open field<br>Use for storage of food items<br>Use for storage of water<br>Use for storage of other pesticides<br>Other, specify: ______________________ | ☐<br>☐<br>☐<br>☐<br>☐<br>☐ |
| 56 | Which of the following practices do you do during spraying pesticides?<br>*Respondent can select more than one option.* | Chew khat<br>Drink water, eat food<br>Smoke cigarette<br>None | ☐<br>☐<br>☐<br>☐ |
| | **Part VII: Preventive measures** | | |
| 57 | Are you trained on the health risks of pesticides, pesticide use, management & application? | Yes<br>No | ☐<br>☐ |
| 58 | During mixing, loading & application, do you read and/or follow the label instructions on pesticide containers? | Yes<br>No | ☐<br>☐ |
| 59 | What measure have you taken when you experienced a symptom presumably due to pesticide exposure/poisoning?<br>*Respondent can select more than one option.* | Visited health facility<br>Used home-based care (drink milk, etc.)<br>Did nothing<br>Other, specify: ________________________ | ☐<br>☐<br>☐ |
| 60 | Do you use any preventive measure during pesticide handling and application? *If the respondent says No, go to Q 62.* | Yes<br>No | ☐<br>☐ |
| 61 | How often do you use the following personal protective equipment during handling and applying a pesticide product? | | |

| | | Always | Sometimes | Never |
|---|---|---|---|---|
| | *Respondent should indicate how often they use personal protective equipment.* | | | |
| | Face mask | ☐ | ☐ | ☐ |
| | Respirator | ☐ | ☐ | ☐ |
| | Goggles or safety glasses | ☐ | ☐ | ☐ |
| | Gloves | ☐ | ☐ | ☐ |
| | Rubber boots | ☐ | ☐ | ☐ |
| | Coveralls | ☐ | ☐ | ☐ |

| | | | |
|---|---|---|---|
| 62 | Do you use any preventive measure other than personal protective equipment during handling and applying a pesticide product? *If no, go to Q64* | Yes<br>No | ☐<br>☐ |
| 63 | Specify preventive measure: ________________________________________________________________ | | |
| 64 | What are the main reasons for not using or rarely using preventive measures? | Not important<br>Very expensive<br>Not available to buy<br>Not comfortable<br>I do not care<br>Other, specify: __________________________ | ☐<br>☐<br>☐<br>☐<br>☐ |
| 65 | Do you change your clothes after the application? | Yes<br>No | ☐<br>☐ |
| 66 | Do you take a shower immediately after finishing spraying? | Always<br>Sometimes<br>Not at all | ☐<br>☐<br>☐ |

***Thank you very much for your cooperation!***
